# No evidence for active viral infection in unicentric and idiopathic multicentric Castleman disease by Viral-Track analysis

**DOI:** 10.1038/s41598-025-85193-x

**Published:** 2025-01-11

**Authors:** Ira Miller, Melanie D. Mumau, Saishravan Shyamsundar, Mateo Sarmiento Bustamante, Pedro Horna, Michael V. Gonzalez, David C. Fajgenbaum

**Affiliations:** 1 Center for Cytokine Storm Treatment & Laboratory, Department of Medicine, University of Pennsylvania, CSTL, 3535 Market Street, Philadelphia, PA 19104 USA; 2 Division of Hematopathology, Mayo Clinic, Rochester, MN 55905 USA

**Keywords:** Castleman disease, Hyperinflammation, Virus detection, Pathogen detection, Viral-Track, Pathogens, Next-generation sequencing

## Abstract

Castleman disease (CD) is a rare hematologic disorder characterized by pathologic lymph node changes and a range of symptoms due to excessive cytokine production. While uncontrolled infection with human herpesvirus-8 (HHV-8) is responsible for the cytokine storm in a portion of multicentric CD (HHV-8-associated MCD) cases, the etiology of unicentric CD (UCD) and HHV-8-negative/idiopathic MCD (iMCD) is unknown. Several hypotheses have been proposed regarding the pathogenesis of UCD and iMCD, including occult infection given the precedent established by HHV-8 infection. To investigate potential active infections in UCD and iMCD, we implemented Viral-Track, a computational method that identifies viral mRNA sequences from next-generation sequencing data. We applied Viral-Track to short sequencing reads from a cohort of UCD (n = 22), iMCD (n = 19), and controls (n = 86). While viral sequences for several unusual viruses were identified in individual CD patients,  sequences for the same virus were not found across multiple CD patients or they were not specific to CD samples and were also found in non-CD samples. These results suggest that active viral infection is unlikely to be a pathological driver of UCD or iMCD.

## Introduction

Castleman disease (CD) comprises a group of inflammatory conditions characterized by enlarged lymph nodes with characteristic histopathological features and a wide range of symptomatology and disease burden. Unicentric CD (UCD) is characterized by a solitary enlarged lymph node with undetectable or mild symptoms; however, in rare cases, UCD can lead to paraneoplastic pemphigus, which is often fatal^1,2^. Multicentric CD (MCD) involves generalized lymphadenopathy and in the most severe cases can lead to multi-organ dysfunction and even death. MCD is further subdivided by etiology: caused by uncontrolled infection with Human Herpesvirus-8 (HHV-8-MCD); caused by neoplastic plasma cells in polyneuropathy, organomegaly, endocrinopathy, monoclonal protein, skin changes (POEMS-MCD); or occurring for an unknown cause in HHV-8-negative/idiopathic MCD (iMCD)^3^. Subclassification of CD is critical for diagnostic, prognostic, and treatment purposes. Like iMCD, UCD also has no known cause. However, in UCD, excision of the enlarged lymph node is often curative with rare recurrence^4–6^. For patients with POEMS-MCD and HHV-8-MCD, treatment is directed at the monoclonal plasma cells and the HHV-8-infected plasmablasts, respectively, and is highly effective^7^. In iMCD, anti-interleukin-6 (IL-6) therapy with siltuximab is effective in approximately one-third to one-half of cases; limited treatment options exist for non-responders^8^. In order to improve the diagnosis and care of patients with iMCD and UCD, a better understanding of the underlying etiology is gravely needed.

Although the pathogenic drivers of UCD and iMCD are unclear, several hypotheses into the mechanistic origin of disease have been proposed including autoimmunity, autoinflammation, malignancy, and infection from an as-yet-identified pathogen^8^. A previous study utilizing virome capture sequencing for vertebrate viruses (VirCapSeq-VERT), a probe-based method that detects all 207 viral taxa known to infect vertebrates with a minimum of 90% sequence homology^9,10^, revealed no evidence of an acute viral infection across a small cohort of UCD and iMCD patients^10^. Although this technology captures a wide breadth of known viruses across approximately 2 million probes, the study had several limitations. The VirCapSeq-VERT system is constrained to detecting only known viruses. Thus, the platform cannot detect novel viruses (with < 75% sequence identity to a known virus). Further, only lymph node tissue was used in the previous study, so evidence of infection in other tissue types would not have been detected. Although this previous study did not detect a previously identified virus, whether iMCD is caused by a novel viral infection has been largely unexplored.

To address the limitations of the previous study, and further investigate the hypothesis that an active viral infection may be present in UCD and iMCD in lymph node and peripheral blood mononuclear cells (PMBCs), we utilized a computational method for detecting viral reads from next-generation sequencing (NGS) data called Viral-Track, that simultaneously aligns NGS reads to both human and viral genomes to identify the presence of mRNA of either human or viral origin^11^. By utilizing Viral-Track, we expanded the number of viruses evaluated from 207 viral taxa to include all viruses, viroid, and satellites published in the NCBI reference sequencing database (~ 10,000)^11^.

Herein, we utilize this method which has not previously been used in the setting of CD to examine whether an acute infection can be identified in CD lymph node tissue or PBMCs and further elucidate possible etiologies of iMCD and UCD.

## Results

### Viral-Track analysis does not identify evidence of shared viral infection in UCD or iMCD

**Fig. 1:**
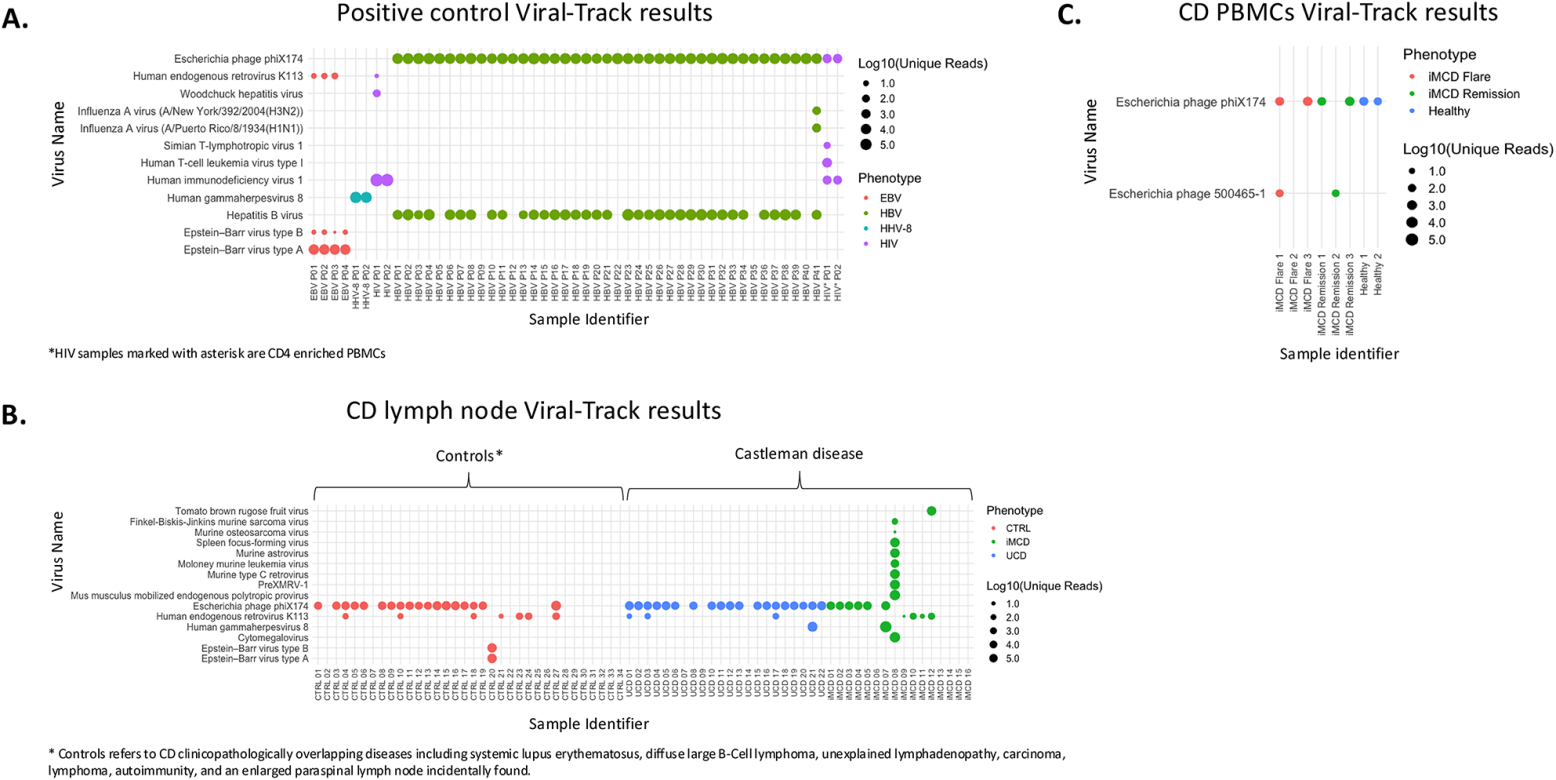
Viral-Track platform successfully detected EBV, HIV, HBV, and HHV-8 positive controls but no clear evidence of an acute infection or shared pathogenic signature was identified in UCD or iMCD samples. Summary of Viral-Track data for (**A**) positive control cohort including samples known to be infected with HIV, HBV, HHV-8, and EBV, (**B**) CD lymph node cohort, (**C)** CD PBMC cohort. The strength of the positive hit was determined by the number of uniquely mapped reads per virus. Positive hits required > 50 mapped reads to a given viral genome, a read complexity threshold > 1.2, and > 10% coverage of the length of the respective viral genome. Abbreviations include Epstein–Barr virus (EBV), Hepatitis B (HBV), Human Herpesvirus 8 (HHV-8), cytomegalovirus (CMV), Human Immunodeficiency virus (HIV), idiopathic multicentric Castleman Disease (iMCD), unicentric Castleman Disease (UCD).

To expand our search of pathogens in CD, we applied the Viral-Track data pipeline to publicly available short-read sequencing-based cohorts and a cohort of iMCD patients and controls we sequenced (Table 1). To determine the ability of Viral-Track to reliably detect viruses in these cohorts, we utilized five datasets of RNA sequencing data from patients with known viral infections (HIV, Epstein-Barr virus (EBV), HHV-8, and Hepatitis B virus [HBV]) that functioned as positive control datasets. In these positive control samples, we were able to detect the respective virus (i.e. HIV, EBV, HBV) in each dataset. Specifically, HIV was detected in 4/4 HIV-infected positive controls, HHV-8 was detected in 1/1 HHV-8-positive controls, EBV was detected in 4/4 EBV positive controls, and HBV was detected in 35/41 HBV positive controls. Utilizing the same analytical parameters for making a positive identification of a viral pathogen within this cohort of positive control samples (n = 50), we also detected short read sequences consistent with six additional viruses including human T-cell leukemia virus type 1 (n = 1), influenza A (n = 1), *Escherichia* phage PhiX174 (n = 43), human endogenous retrovirus K113 (n = 4), woodchuck hepatitis virus (n = 1), and simian T-lymphotropic virus 1 (n = 1) (Fig. 1A).

**Table 1:** Summary of Viral-Track cohorts.

Cohort	Tissue source and description	Phenotype	Positve Control	Control virus detected (# pos/total)	Sequencing type (Bulk/Single-cell)	Accession number (subset)	Citation
**1**	FFPE lymph node biopsy slides	9 iMCD	N	–	Bulk	GSE263321	–
		15 Controls*					
**2**	Fresh-frozen lymph node tissue	7 iMCD	N	–	Bulk	GSE195477	17
		22 UCD					
		19 Controls*					
**3**	PBMCs	3 iMCD	N	–	Single-cell	GSE140881	18
		2 HD					
**4**	Healthy donor PBMCs incubated with EBV	4 EBV	Y	4/4	Single-cell	E-MTAB-7805 (SRR16976504, SRR16976505, SRR16976520, SRR16976521)	19
**5**	FFPE core needle liver biopsy	41 HBV	Y	35/41	Bulk	GSE230397 (SRR24310176: SRR24310216)	20
**6**	Primary effusion lymphoma derived cell line cells incubated with HHV-8	1 HHV-8	Y	1/1	Single-cell	GSE154900	21
**7**	Healthy donor PBMCs incubated with HIV	2 HIV	Y	2/2	Single-cell	SAMN08685502 SAMN08685501	22
**8**	CD4 enriched PBMCs	2 HIV	Y	2/2	Single-cell	GSE187515	23

*Controls refers to CD clinicopathologically overlapping diseases including systemic lupus erythematosus, diffuse large B-Cell lymphoma, unexplained lymphadenopathy, carcinoma, lymphoma, autoimmunity, and an enlarged paraspinal lymph node incidentally found. HD, healthy donor.

To determine if CD patient samples with unknown infection status had evidence of viral infection using Viral-Track, we again applied the same analytical parameters used in the positive control cohort to three RNA sequencing datasets from CD patients with unknown infection status (iMCD, n = 19 [16 FFPE LN, 3 PMBC]; UCD, n = 22; related lymphadenopathies, n = 34; healthy donors [assumed negative controls], n = 2) (**Table S1**). First, we evaluated RNA sequencing data from lymph node tissue in CD patients with unknown infection status (iMCD, n = 16; UCD n = 22). Overall, we identified evidence of 13 viruses in iMCD FFPE LN samples, and 3 in UCD FFPE LN samples (Table 2). While evidence for several unusual pathogens was identified in individual UCD or iMCD patients, none of these viruses were found across multiple CD patients (i.e., Tomato brown rugose fruit virus, Finkel-Biskis-Jinkins murine sarcoma virus, murine osteosarcoma virus, spleen focus-forming virus, murine astrovirus, Moloney murine leukemia virus, murine type C retrovirus, PreXMRY-1, mus musculus mobilized endogenous polytropic provirus), or identified viruses were not specific to CD samples and were also found in non-CD samples (*Escherichia* phage phix174, human endogenous retrovirus K113) (Fig. 1B). Interestingly, one UCD and one iMCD sample each from the CD cohort showed evidence of HHV-8 infection and one iMCD sample from a patient with multiple unusual viruses detected (iMCD 08) was found to have evidence of cytomegalovirus (CMV) infection. Retrospective review of LANA-1 immunohistochemistry (IHC) of lymph node tissue for HHV-8 failed to reveal evidence of HHV-8 in either HHV-8-detected CD cases (**Figure S1**). Whether these cases truly have HHV-8 driven CD, this represents detection of a bystander infection, or there is another explanation for the discrepancy in HHV-8 detection was difficult to ascertain as clinical information was limited. In the CMV-positive iMCD patient, retrospective analysis of clinical records did identify evidence of a previous CMV infection (IgG + /IgM-/PCR-) 12 and 20 days prior to the biopsy with evidence of CMV by IgG antibody test (**Table S2**).

After finding no strong evidence of shared acute viral infection in CD lymph node tissue, we investigated whether evidence of a pathogenic virus could be identified in PBMCs of iMCD patients (Fig. 1C). We tested six PBMC samples from three iMCD patients in active disease (flare) and remission with unknown infection status as well as two healthy controls, that functioned as negative controls. Only two innocuous *Escherichia* phages, phiX174 and 500,465–1, were identified at low RNA levels in these cases^13^. 

In all, 14 viruses were identified in either iMCD or UCD samples. To evaluate if any of the 14 viruses with sequences detected at a significantly higher frequency in CD samples than controls, we compared the proportion of positive cases between CD cohorts and healthy controls (Table 2). Of the 14 viruses identified in CD samples, no virus was detected at a significantly higher proportion in CD compared to controls (Table 2). Taken together, our analyses were unable to detect a common virus shared across CD patients and suggests that active viral infection is an unlikely cause of UCD and iMCD.

**Table 2:** Comparison between CD samples and relevant controls for viruses identified in tissue from CD patients.

Tissue type	Phenotype	Virus name	CD positive proportion (#positive/total)	Controls positive * ratio (#positive/total)	p.value	p.adj
**Lymph node**	iMCD	Human gammaherpesvirus 8	1/16	0/34	0.72	1.00
		Tomato brown rugose fruit virus	1/16	0/34	0.72	1.00
		Mus musculus mobilized endogenous polytropic provirus	1/16	0/34	0.72	1.00
		Cytomegalovirus	1/16	0/34	0.72	1.00
		PreXMRV-1	1/16	0/34	0.72	1.00
		Murine type C retrovirus	1/16	0/34	0.72	1.00
		Moloney murine leukemia virus	1/16	0/34	0.72	1.00
		Murine astrovirus	1/16	0/34	0.72	1.00
		Spleen focus-forming virus	1/16	0/34	0.72	1.00
		Murine osteosarcoma virus	1/16	0/34	0.72	1.00
		Finkel-Biskis-Jinkins murine sarcoma virus	1/16	0/34	0.72	1.00
		*Escherichia* phage phiX174	6/16	18/34	0.73	1.00
		Human endogenous retrovirus K113	4/16	7/34	1.00	1.00
	UCD	*Escherichia* phage phiX174	21/22	18/34	0.23	0.70
		Human endogenous retrovirus K113	3/22	7/34	0.84	1.00
		Human gammaherpesvirus 8	1/22	0/34	0.84	1.00
**PBMC**	iMCD flare	*Escherichia* phage 500,465–1	1/3	0/2	1.00	1.00
		*Escherichia* phage phiX174	2/3	2/2	1.00	1.00
	iMCD Remission	*Escherichia* phage 500,465–1	1/3	0/2	1.00	1.00
		*Escherichia* phage phiX174	2/3	2/2	1.00	1.00

*Controls refers to CD clinicopathologically overlapping diseases including systemic lupus erythematosus, diffuse large B-Cell lymphoma, unexplained lymphadenopathy, carcinoma, lymphoma, autoimmunity, and an enlarged paraspinal lymph node incidentally found.

## Discussion

To date, data to determine a pathogenic driver of iMCD has been limited. Previously, VirCapSeq-VERT, a probe-based method was used to search for known viral taxa in a limited cohort of CD samples, which was unable to identify a known acute virus in CD^10^. To our knowledge, this is the only report of an investigation into a potential infectious etiology in UCD or iMCD. The Viral-Track platform used in the present study has been previously used to identify pathogens in diverse tissue samples and disease states, including severe acute respiratory syndrome coronavirus 2 (SARS-CoV-2), influenza, HIV, and HBV, among others^11,14,15^. This study represents the largest effort to date for evaluating potential viral infections in UCD (n = 22) and iMCD (n = 19). Importantly, Viral-Track demonstrated the ability to correctly identify pathogens in the positive control cohorts used: Viral-Track successfully detected HIV, HHV-8, EBV, and HBV in known infected samples. Although this approach was capable of identifying pathogens in positive controls, we did not detect evidence of a virus in the lymph node or blood of multiple UCD or iMCD patients, which was not present in controls, that would be suggestive of an active infection driving UCD or iMCD pathology.

Utilizing the Viral-Track platform, there were several unexpected viruses identified in the positive control cohort (human T-cell leukemia virus type 1, influenza A, *Escherichia *phage phiX174, human endogenous retrovirus K113, woodchuck hepatitis virus, and simian T-lymphotropic virus 1). There was no indication of infection by human T-cell leukemia virus and influenza A virus in each patient’s respective clinical records; however, these viruses are species-appropriate, and infection is clinically feasible^16,17^. *Escherichia* phage phiX174 and human endogenous retrovirus K113 were detected in a variety of CD patient samples, healthy controls, and positive controls with no discernable pattern. The detection of *Escherichia *phage phiX174, an innocuous bacteria phage, is often attributed to optical errors from PHIX libraries^18^. The presence of human endogenous retrovirus K113 was also not surprising as 30% of the human population carries the K113 retroviral genome and it is actively transcribed^19^. Thus, these unexpected viruses with sequences identified are unlikely to be playing a role in the positive controls.

In the CD Viral-Track cohort, sequences from 14 infectious agents were detected, including *Escherichia *phage phiX174 bacteria phage and human endogenous retrovirus K113. Nine other viruses known to infect non-human hosts but not humans (tomato brown rugose fruit virus, Mus musculus mobilized endogenous polytropic provirus, PreXMRV-1, Murine type C retrovirus, Moloney Murine leukemia virus, Murine astrovirus, spleen focus-forming virus, Murine osteosarcoma virus, and Finkel-Biskis-Jinkins murine sarcoma virus^20–28^) were also detected in the CD cohort. Of note, 8/9 of these were detected in the same sample from a patient who also had evidence of CMV infection. Importantly, none of these non-human-host viruses were identified in multiple CD samples.

Of potential clinical interest was the detection of HHV-8 mRNA in a UCD and an iMCD patient who both had LANA-1 negative staining for HHV-8 and were being treated as HHV-8-negative CD (**Figure S1**). When treating CD, determining HHV-8 status is critical for care as rituximab is highly effective for HHV-8-MCD, but not iMCD^8,29^. A study by Hammock et al. suggests that LANA-1 staining may not be sensitive enough to identify HHV-8 in all HHV-8-positive samples. While LANA-1 staining is specific, it is not as sensitive as other nucleotide-based methods such as PCR. By comparing LANA-1 IHC to PCR in HHV-8 + sarcoma (n = 24), the LANA-1 stain by IHC missed HHV-8 in 2/24 (8.3%) samples while the PCR method detected HHV-8 in all samples^30^. As HHV-8 was only identified in two LANA-1 negative CD patients, one with UCD and the other with presumed iMCD, confirmation of HHV-8 infection by PCR could potentially clarify the true infection status and improve patient care. In addition to running PCR to confirm HHV-8 status, it would be interesting to determine if either of these 2 patients had similar clinical presentations to HHV-8-MCD patients, however, due to the de-identification of clinical samples, clinical data in these cases was limited and prevented further evaluation. It is important to note that the LANA-1 negative (HHV-8 positive by Viral-Track) samples in this study were not tested by PCR. However, within RNA-sequencing protocols, there are PCR amplification steps during library preparation^31^, potentially highlighting the superiority of PCR detection of HHV-8 compared to LANA-1 staining. Also of interest was the detection of another herpesvirus, CMV, by Viral-Track in one iMCD lymph node sample. Upon retrospective analysis of clinical records, evidence of a prior CMV infection (IgG + /IgM-/PCR-) was identified from clinical testing of peripheral blood. However, as it was only detected in one CD sample and clinical testing suggested it represented a prior infection, CMV is unlikely to be the driver of CD in this patient.

There are several limitations to this work. First, the results from our investigation of pathogens in UCD and iMCD presented in this study do not completely rule out the possibility that a microorganism is involved in the pathogenesis of UCD or iMCD. Molecular mimicry, as described in long-COVID, rheumatic fever, post-streptococcal glomerulonephritis, and Guillain Barré syndrome, is one possible mechanism through which prior infection with a pathogen could cause iMCD or UCD but not be present at detectable levels in the lymph node or blood^32^. In these diseases, the infection does not coincide with the symptoms temporally or spatially (i.e. the infection takes place before the specific symptoms and in a different location than the affected organs). Although it is reasonable to assume that the pathogen would be detectable in secondary lymph node organs and/or in the blood as is the case in HHV-8-MCD, it is also possible that the acute infection may be present elsewhere such as in the gastrointestinal tract, lungs, bone marrow, or liver. Second, the search for microorganisms in iMCD and UCD is very challenging given that CD is a rare disease and tissue is limited, which may have resulted in this study being underpowered to detect pathogens rarely present across CD patients. Third, some known viruses may not have been able to be captured in our analysis. Not all viruses such as lymphocytic choriomeningitis virus synthesize mRNA with poly(A) tails which are required for RNA capture technologies^11^. Future experiments using random primers to detect these RNA transcripts without poly(A) tails are warranted. Fourth, many novel viruses would go undetected as Viral-Track is only able to identify known viruses with at least 75–90% homology to known viruses. Finally, the relatively small number of viral reads relative to human reads from bulk tissue or the relatively limited sequencing depth of single cell tissue may have limited the ability to detect sequences from viral pathogens, if present.  

Taken together, the results of this study provide further insight into the etiology of UCD and iMCD, suggesting that an acute viral infection is an unlikely cause. Although the search and characterization of pathogens in CD patients should not be completely ruled out, our results indicate that other hypothesized etiologies of iMCD and UCD should be prioritized. Future investigations into the mechanism underlying CD such as auto/neoantigens, somatic and germline mutations, and autoinflammation will provide further insights into potential etiologies of these diseases.

## Materials and methods

### Patient materials

The collection of samples was approved by local ethics committee, where applicable, and by the University of Pennsylvania institutional review board^33^. In addition, all methods performed in this study were performed in accordance with relevant guidelines and regulations. All patients provided informed consent for the collection and use of their tissue. All patients satisfied the international consensus diagnostic clinical criteria for UCD or iMCD^3,34^. Briefly, clinical information was collected from ACCELERATE, a natural history registry of CD (ClinicalTrials.gov identifier: NCT02817997, first posted date: 06/29/2016)^33^. The eligibility criteria to enroll into ACCELERATE requires a pathology report suggestive of CD^33^. Once a patient is enrolled, clinical, radiological, and laboratory data from medical records are extracted into ACCELERATE by trained data analysts. After data compilation, patients are evaluated by a panel of expert clinicians and hematopathologists, who review and adjudicate each case based on all available information to determine the relative likelihood of a CD diagnosis. Diagnostic clinical characteristics are summarized for each cohort (Table 3).

**Table 3:** Clinical data for CD patients by cohort.

	**Viral-Track iMCD Lymph Node**	Viral-Track iMCD PBMC*
Number of samples	9	3
Tissue	FFPE lymph node	PBMC
Age at diagnosis, in years Mean (SD)	49.1 (12)	38.7 (11.6)
Sex (M:F)	4:5	3:0
Thrombocytopenia (present/assessed)	7/9	2/3
Anasarca or edema (present/assessed)	8/8	2/3
Constitutional symptoms (present/assessed)	7/8	3/3
Renal dysfunction (present/assessed)	7/8	2/3
Organomegaly (present/assessed)	6/8	2/3
CRP (mg/L)	104.6 (113.6), n = 6	178.8 (115.3), n = 2
ESR (mm/hr)	76.6 (25.4), n = 7	72.7 (44.6), n = 3
Hemoglobin (g/dL)	8.4 (2.8), n = 8	6.7 (0.1), n = 3
Albumin (g/dL)	2.5 (1.1), n = 8	2.1 (0.8), n = 3
Creatinine (mg/dL)	2 (0.8), n = 8	2.2 (1.7), n = 3

*Data from Pai, R. L. et al. Type I IFN response associated with mTOR activation in the TAFRO subtype of idiopathic multicentric Castleman disease. JCI Insight 5 (2020). For clinical data from the other CD samples evaluated please see Horna, P., et al. The lymph node transcriptome of unicentric and idiopathic multicentric Castleman disease. Haematologica (2022). All clinical laboratory values above are reported as Mean(SD).

### Viral-Track cohort selection

Cohorts of eight clinically distinct entities were obtained from various publicly available databases as outlined in Table 1. Three of the eight cohorts had NGS data derived from CD samples and other comparator disease groups, including **(1)** Formalin-fixed paraffin-embedded (FFPE) lymph node tissue from iMCD (n = 9), systemic lupus erythematosus (SLE = 3), diffuse large B-cell lymphoma (DLBCL, n = 5), reactive lymph nodes with inconclusive pathology (reactive, n = 7) patients, **(2) **fresh-frozen lymph node tissue from UCD (n = 22), iMCD (n = 7), reactive lymph nodes with inconclusive pathology (n = 10), carcinoma (n = 3), lymphoma (n = 3), autoimmunity (n = 2), and an enlarged paraspinal lymph node incidentally found during surgery (n = 1)^35^, **(3)** matched fresh-frozen PBMCs from iMCD patients during flare (n = 3) and remission (n = 3) disease states and healthy controls (n = 2) (Table 1**, Table S1**)^36^. Importantly, all non-CD samples that were not explicitly positive control samples were assumed to be negative controls for active infection. The remaining five cohorts were included as positive controls for Viral-Track analysis: **(4) **PBMCs infected with EBV (n = 4)^37^, **(5) **Hepatitis B (HBV) positive FFPE core needle biopsies (n = 41)^38^, **(6) **primary effusion lymphoma (PEL) cell lines infected with HHV-8 (n = 1)^39^, **(7) **PMBCs infected ex vivo with human immunodeficiency virus (HIV) (n = 2)^40^, **(8) **CD4 enriched PBMCs from HIV patients (n = 2)^41^.

### Immunohistochemistry protocol and LANA-1 staining

Formalin-fixed/paraffin-embedded tissue sections were immunostained on a Benchmark ULTRA autostainer (Roche Diagnostics, Indianapolis, IN, USA), using a primary anti-HHV-8 latent nuclear antigen-1 (LANA-1) mouse monoclonal antibody (clone 13B10, catalog #: 265 M-14-ASR; Cell Marque Corporation, Rocklin, CA; USA).

### Viral-Track analysis

Viral-Track is a computational pipeline that utilizes the STAR aligner to identify next-generation sequencing (NGS) reads that map to viral genomes, as previously described in detail^11,42^. Briefly, fastq files were downloaded using the SRA Toolkit from NCBI and formatted with the UMI tools per the Viral-Track protocol^11,43^. GRCh38 human reference (10 × Genomics Human reference GRCh38 version 2020-A) and viruSITE database (viruSITE, release 2023.2, Stano, et al. 2016, http://www.virusite.org/index.php) were downloaded from the viruSITE and 10X genomic website and used to build a STAR index with a genomeSAindexNbases value of 13 and a geomeChrBinNbits value of 18^42^. The STAR aligner (version 2.7.4a) was used to simultaneously align sequencing reads to human reference and viral genomes with the recommended setting of Viral-Track (https://github.com/PierreBSC/Viral-Track)^11^. Several quality control steps were taken to minimize non-specific mapping. A positive viral hit required > 50 uniquely mapped sequencing reads to a given viral genome, a mean read complexity threshold > 1.2, and > 10% coverage of the length of the respective viral genome; these are the standard parameters for a positive hit in Viral-Track. Any virus exceeding these thresholds was considered a positive hit and explored further.

## Supplementary Information

Supplementary Information 1.

Supplementary Information 2.

## Data Availability

The datasets generated during and/or analyzed in this study are available in the GEO repository https://www.ncbi.nlm.nih.gov/geo/query/acc.cgi?acc = GSE263321. The accession number for the data reported in this paper is GEO:GSE263321. All scripts used to analyze and create figures for this manuscript can be found in the following github repository: https://github.com/UPenn-CSTL.

## References

[1] Sarmiento Bustamante, M. et al. Ongoing symptoms following complete surgical excision in unicentric Castleman disease. *Am. J. Hematol.* **98**, E334–E337 (2023). 37635628 10.1002/ajh.27065 PMC10998479

[2] Dieudonné, Y. et al. Paraneoplastic pemphigus uncovers distinct clinical and biological phenotypes of western unicentric Castleman disease. *Br. J. Haematol.*10.1111/bjh.18847 (2023). 37221131 10.1111/bjh.18847

[3] Fajgenbaum, D. C., Uldrick, T. S. & Bagg, A. International, evidence-based consensus diagnostic criteria for HHV-8–negative/idiopathic multicentric Castleman disease. *Blood J.* **129**(12), 1646–1657 (2017). 10.1182/blood-2016-10-746933 PMC5364342 28087540

[4] Chronowski, G. M. et al. Treatment of unicentric and multicentric Castleman disease and the role of radiotherapy. *Cancer* **92**, 670–676 (2001). 11505414 10.1002/1097-0142(20010801)92:3<670::aid-cncr1369>3.0.co;2-q

[5] Boutboul, D. et al. Treatment and outcome of unicentric Castleman disease: a retrospective analysis of 71 cases. *Br. J. Haematol.* **186**, 269–273 (2019). 31016730 10.1111/bjh.15921

[6] Oksenhendler, E. et al. The full spectrum of Castleman disease: 273 patients studied over 20 years. *Br. J. Haematol.* **180**, 206–216 (2018). 29143319 10.1111/bjh.15019

[7] Gerard, L. et al. Prospective study of rituximab in chemotherapy-dependent human immunodeficiency virus associated multicentric Castleman’s disease: ANRS 117 CastlemaB Trial. *J. Clin. Oncol.* **25**, 3350–3356 (2007). 17664482 10.1200/JCO.2007.10.6732

[8] Pierson, S. K. et al. Treatment consistent with idiopathic multicentric Castleman disease guidelines is associated with improved outcomes. *Blood Adv.* **7**, 6652–6664 (2023). 37656441 10.1182/bloodadvances.2023010745 PMC10637880

[9] Briese, T. et al. Virome capture sequencing enables sensitive viral diagnosis and comprehensive virome analysis. *MBio*10.1128/mBio.01491-15 (2015). 26396248 10.1128/mBio.01491-15 PMC4611031

[10] Nabel, C. S. et al. Virome capture sequencing does not identify active viral infection in unicentric and idiopathic multicentric Castleman disease. *PLoS One* **14**, e0218660 (2019). 31242229 10.1371/journal.pone.0218660 PMC6594611

[11] Bost, P. et al. Host-Viral infection maps reveal signatures of severe COVID-19 Patients. *Cell* **181**, 1475-1488.e12 (2020). 32479746 10.1016/j.cell.2020.05.006 PMC7205692

[12] Zhang, M.-Y. et al. UCD with MCD-like inflammatory state: surgical excision is highly effective. *Blood Adv.* **5**, 122–128 (2021). 33570636 10.1182/bloodadvances.2020003607 PMC7805307

[13] Shimasaki, N., Okaue, A., Kikuno, R. & Shinohara, K. Comparison of the filter efficiency of medical nonwoven fabrics against three different microbe aerosols. *Biocontrol Sci.* **23**, 61–69 (2018). 29910210 10.4265/bio.23.61

[14] Kim, S. C. et al. Efficacy of antiviral therapy and host-virus interactions visualised using serial liver sampling with fine-needle aspirates. *JHEP Rep.* **5**, 100817 (2023). 37600958 10.1016/j.jhepr.2023.100817 PMC10432215

[15] Seckar, T. et al. Detection of microbial agents in oropharyngeal and nasopharyngeal samples of SARS-CoV-2 patients. *Front. Microbiol.* **12**, 637202 (2021). 33790878 10.3389/fmicb.2021.637202 PMC8006406

[16] Matsuoka, M. & Jeang, K. T. Human T-cell leukaemia virus type 1 (HTLV-1) infectivity and cellular transformation. *Nat. Rev. Cancer* **7**, 270–280 (2007). 17384582 10.1038/nrc2111

[17] Javanian, M. et al. A brief review of influenza virus infection. *J. Med. Virol.* **93**, 4638–4646 (2021). 33792930 10.1002/jmv.26990

[18] Forster, M. et al. Vy-PER: eliminating false positive detection of virus integration events in next generation sequencing data. *Sci. Rep.* **5**, 11534 (2015). 26166306 10.1038/srep11534 PMC4499804

[19] Beimforde, N., Hanke, K., Ammar, I., Kurth, R. & Bannert, N. Molecular cloning and functional characterization of the human endogenous retrovirus K113. *Virology* **371**, 216–225 (2008). 18076964 10.1016/j.virol.2007.09.036

[20] Salem, N. M., Jewehan, A., Aranda, M. A. & Fox, A. Tomato brown rugose fruit virus pandemic. *Annu. Rev. Phytopathol.* **61**, 137–164 (2023). 37268006 10.1146/annurev-phyto-021622-120703

[21] Boi, S. et al. Endogenous retroviruses mobilized during friend murine leukemia virus infection. *Virology* **499**, 136–143 (2016). 27657834 10.1016/j.virol.2016.07.009 PMC5102782

[22] Delviks-Frankenberry, K. et al. Generation of multiple replication-competent retroviruses through recombination between PreXMRV-1 and PreXMRV-2. *J. Virol.* **87**, 11525–11537 (2013). 23966380 10.1128/JVI.01787-13 PMC3807343

[23] Scolnick, E. M. & Parks, W. P. Harvey sarcoma virus: a second murine type C sarcoma virus with rat genetic information. *J. Virol.* **13**, 1211–1219 (1974). 4364897 10.1128/jvi.13.6.1211-1219.1974 PMC355440

[24] Kuchino, Y., Beier, H., Akita, N. & Nishimura, S. Natural UAG suppressor glutamine tRNA is elevated in mouse cells infected with Moloney murine leukemia virus. *Proc. Natl. Acad. Sci. U. S. A.* **84**, 2668–2672 (1987). 3472229 10.1073/pnas.84.9.2668 PMC304719

[25] Morita, H. et al. Pathogenesis of murine astrovirus in experimentally infected mice. *Exp. Anim.* **70**, 355–363 (2021). 33828018 10.1538/expanim.20-0162 PMC8390316

[26] Clarke, B. J., Axelrad, A. A. & Housman, D. Friend spleen focus-forming virus production in vitro by a nonerythroid cell line. *J. Natl. Cancer Inst.* **57**, 853–859 (1976). 1069858 10.1093/jnci/57.4.853

[27] Curran, T., Peters, G., Van Beveren, C., Teich, N. M. & Verma, I. M. FBJ murine osteosarcoma virus: identification and molecular cloning of biologically active proviral DNA. *J. Virol.* **44**, 674–682 (1982). 6292525 10.1128/jvi.44.2.674-682.1982 PMC256311

[28] Pavković, Ž et al. Brain molecular changes and behavioral alterations induced by propofol anesthesia exposure in peripubertal rats. *Paediatr. Anaesth.* **27**, 962–972 (2017). 28772011 10.1111/pan.13182

[29] Uldrick, T. S. et al. Rituximab plus liposomal doxorubicin in HIV-infected patients with KSHV-associated multicentric Castleman disease. *Blood* **124**, 3544–3552 (2014). 25331113 10.1182/blood-2014-07-586800 PMC4256906

[30] Hammock, L. et al. Latency-associated nuclear antigen expression and human herpesvirus-8 polymerase chain reaction in the evaluation of kaposi sarcoma and other vascular tumors in HIV-positive patients. *Mod. Pathol.* **18**, 463–468 (2005). 15578080 10.1038/modpathol.3800221

[31] Parekh, S., Ziegenhain, C., Vieth, B., Enard, W. & Hellmann, I. The impact of amplification on differential expression analyses by RNA-seq. *Sci. Rep.* **6**, 25533 (2016). 27156886 10.1038/srep25533 PMC4860583

[32] Rodriguez, Y. et al. Guillain-Barre syndrome, transverse myelitis and infectious diseases. *Cell. Mol. Immunol.* **15**, 547–562 (2018). 29375121 10.1038/cmi.2017.142 PMC6079071

[33] Pierson, S. K. et al. ACCELERATE: A patient-powered natural history study design enabling clinical and therapeutic discoveries in a rare disorder. *Cell. Rep. Med.* **1**, 100158 (2020). 33377129 10.1016/j.xcrm.2020.100158 PMC7762771

[34] van Rhee, F. et al. International evidence-based consensus diagnostic and treatment guidelines for unicentric Castleman disease. *Blood Adv.* **4**, 6039–6050 (2020). 33284946 10.1182/bloodadvances.2020003334 PMC7724917

[35] Horna, P., King, R. L., Jevremovic, D., Fajgenbaum, D. C. & Dispenzieri, A. The lymph node transcriptome of unicentric and idiopathic multicentric Castleman disease. *Haematologica*10.3324/haematol.2021.280370 (2022). 10.3324/haematol.2021.280370 PMC9827154 35484648

[36] Pai, R.-A.L. et al. Type I IFN response associated with mTOR activation in the TAFRO subtype of idiopathic multicentric Castleman disease. *JCI Insight*10.1172/jci.insight.135031 (2020). 32376796 10.1172/jci.insight.135031 PMC7253017

[37] Mrozek-Gorska, P. et al. Epstein-Barr virus reprograms human B lymphocytes immediately in the prelatent phase of infection. *Proc. Natl. Acad. Sci. U S A* **116**, 16046–16055 (2019). 31341086 10.1073/pnas.1901314116 PMC6690029

[38] Montanari, N. R. et al. Multi-parametric analysis of human livers reveals variation in intrahepatic inflammation across phases of chronic hepatitis B infection. *J. Hepatol.* **77**, 332–343 (2022). 35218813 10.1016/j.jhep.2022.02.016

[39] Rondeau, N. C., Finlayson, M. O. & Miranda, J. L. Widespread Traces of Lytic Kaposi Sarcoma-Associated Herpesvirus in Primary Effusion Lymphoma at Single-Cell Resolution. *Microbiol. Resour. Announc.*10.1128/MRA.00851-20 (2020). 33154001 10.1128/MRA.00851-20 PMC7645656

[40] Bradley, T., Ferrari, G., Haynes, B. F., Margolis, D. M. & Browne, E. P. Single-cell analysis of quiescent hiv infection reveals host transcriptional profiles that regulate proviral latency. *Cell Rep.*10.1016/j.celrep.2018.09.020 (2018). 30282021 10.1016/j.celrep.2018.09.020 PMC6258175

[41] Collora, J. A. et al. Single-cell multiomics reveals persistence of HIV-1 in expanded cytotoxic T cell clones. *Immunity* **55**, 1013-1031.e7 (2022). 35320704 10.1016/j.immuni.2022.03.004 PMC9203927

[42] Dobin, A. et al. STAR: ultrafast universal RNA-seq aligner. *Bioinformatics* **29**, 15–21 (2013). 23104886 10.1093/bioinformatics/bts635 PMC3530905

[43] Smith, T., Heger, A. & Sudbery, I. UMI-tools: modeling sequencing errors in Unique Molecular Identifiers to improve quantification accuracy. *Genome Res.* **27**, 491–499 (2017). 28100584 10.1101/gr.209601.116 PMC5340976

