# Supplementary figures and images for: No evidence for active viral infection in unicentric and idiopathic multicentric Castleman disease by Viral-Track analysis
Source: Sci Rep. 2025 Jan 11;15:1676. doi: 10.1038/s41598-025-85193-x (PMC11724840; doi:10.1038/s41598-025-85193-x)

**Figure S1: LANA-1 stain for RNA HHV-8 positive CD patients**

**UCD P17**

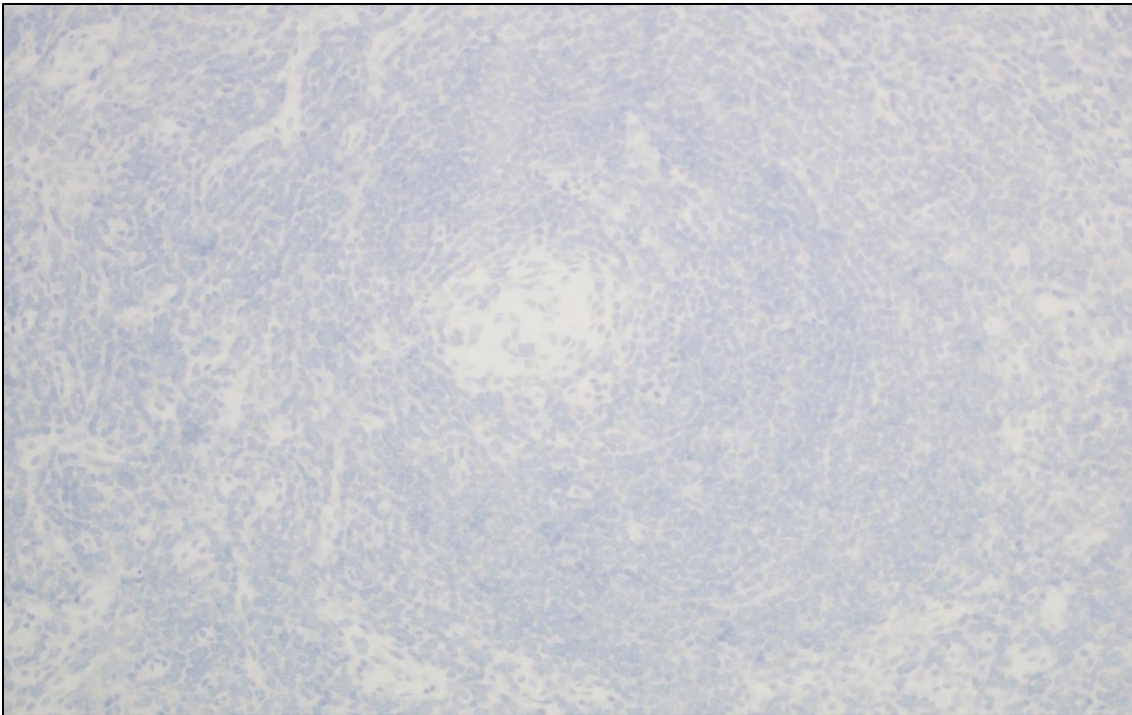

**iMCD P07**

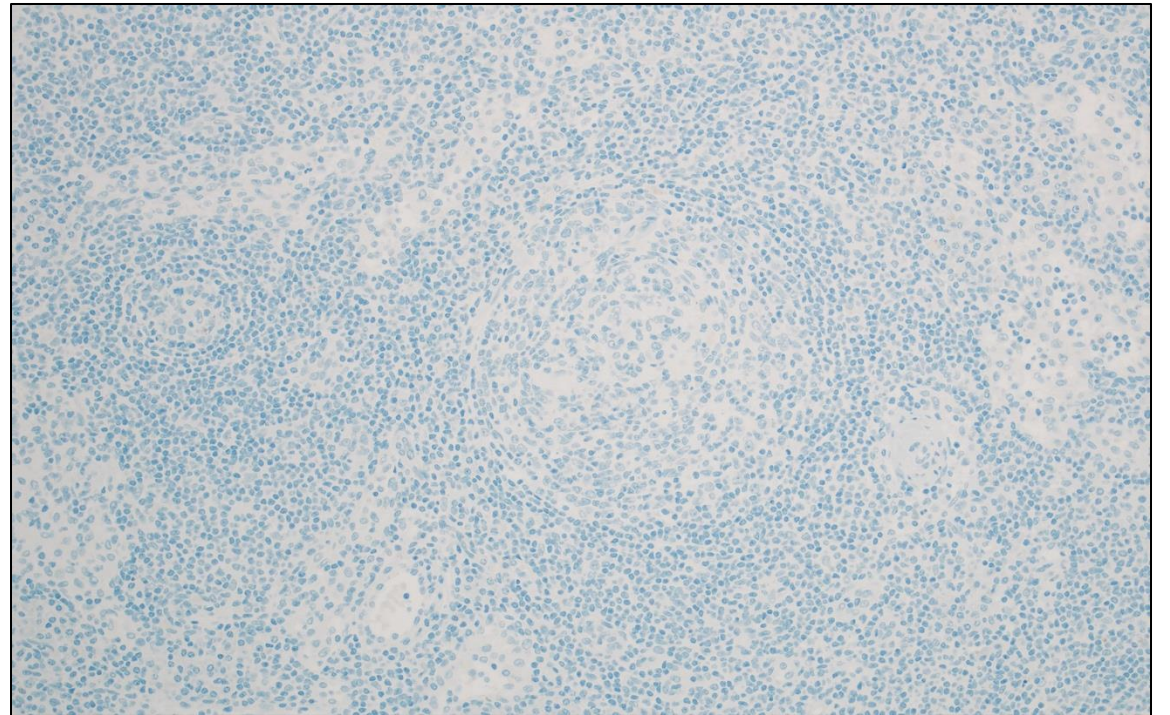

Supplement: Supplementary file 1 — Supplementary Information 1. [file 41598_2025_85193_MOESM1_ESM.pdf]
