# Supplementary material for: No evidence for active viral infection in unicentric and idiopathic multicentric Castleman disease by Viral-Track analysis
Source: Sci Rep. 2025 Jan 11;15:1676. doi: 10.1038/s41598-025-85193-x (PMC11724840; doi:10.1038/s41598-025-85193-x)
Supplement: Supplementary file 2 — Supplementary Information 2. [file 41598_2025_85193_MOESM2_ESM.docx]

| Table S1. Meta data for control lymph nodes | | |
| --- | --- | --- |
| Sample name | **Phenotype** | **Institution of origin** |
| CTRL 1 | Lymphoma | Mayo Clinic |
| CTRL 2 | Reactive LN with uncertain histology | Mayo Clinic |
| CTRL 3 | Reactive LN with uncertain histology | Mayo Clinic |
| CTRL 4 | Reactive LN with uncertain histology | Mayo Clinic |
| CTRL 5 | Lymphoma | Mayo Clinic |
| CTRL 6 | Lymphoma | Mayo Clinic |
| CTRL 7 | Reactive LN with uncertain histology | Mayo Clinic |
| CTRL 8 | History of Lymphoma | Mayo Clinic |
| CTRL 9 | History of Autoimmune diseases | Mayo Clinic |
| CTRL 10 | Carcinoma | Mayo Clinic |
| CTRL 11 | Reactive LN with uncertain histology | Mayo Clinic |
| CTRL 12 | Reactive LN with uncertain histology | Mayo Clinic |
| CTRL 13 | Reactive LN with uncertain histology | Mayo Clinic |
| CTRL 14 | Autoimmune | Mayo Clinic |
| CTRL 15 | Orthapedic surgery | Mayo Clinic |
| CTRL 16 | Reactive LN with uncertain histology | Mayo Clinic |
| CTRL 17 | Carcinoma | Mayo Clinic |
| CTRL 18 | Reactive LN with uncertain histology | Mayo Clinic |
| CTRL 19 | Carcinoma | Mayo Clinic |
| CTRL 20 | Diffuse large B-cell lymphoma | University of Pennsylvania |
| CTRL 21 | Reactive LN with uncertain histology | University of Pennsylvania |
| CTRL 22 | Reactive LN with uncertain histology | University of Pennsylvania |
| CTRL 23 | Diffuse large B-cell lymphoma | University of Pennsylvania |
| CTRL 24 | Diffuse large B-cell lymphoma | University of Pennsylvania |
| CTRL 25 | Diffuse large B-cell lymphoma | University of Pennsylvania |
| CTRL 26 | Diffuse large B-cell lymphoma | University of Pennsylvania |
| CTRL 27 | Systemic lupus erythematosus | University of Pennsylvania |
| CTRL 28 | Reactive LN with uncertain histology | University of Pennsylvania |
| CTRL 29 | Reactive LN with uncertain histology | University of Pennsylvania |
| CTRL 30 | Reactive LN with uncertain histology | University of Pennsylvania |
| CTRL 31 | Systemic lupus erythematosus | University of Pennsylvania |
| CTRL 32 | Systemic lupus erythematosus | University of Pennsylvania |
| CTRL 33 | Reactive LN with uncertain histology | University of Pennsylvania |
| CTRL 34 | Reactive LN with uncertain histology | University of Pennsylvania |

| Table S2. CMV associated tests for CMV positive iMCD patient | | | |
| --- | --- | --- | --- |
| Days before biopsy | IgG | IgM | PCR |
| 20 | Pos | Neg | Neg |
| 12 | Pos | Neg | Neg |
